# Supplementary material for: Identification and functional analysis of non-coding regulatory small RNA FenSr3 in Bacillus amyloliquefaciens LPB-18
Source: PeerJ. 2023 May 15;11:e15236. doi: 10.7717/peerj.15236 (PMC10194069; doi:10.7717/peerj.15236)
Supplement: Supplemental Information 4 [file peerj-11-15236-s004.zip › KO/CK-vs-T1_map/map04212.html]

KEGG PATHWAY: Longevity regulating pathway - worm - Reference pathway


|  |  |
| --- | --- |
| **Longevity regulating pathway - worm - Reference pathway** |  |

[
Pathway menu
| Organism menu
| Pathway entry
| Show description
| User data mapping
]

The nematode Caenorhabditis elegans has proven to be a very useful tool for studying the genetics of longevity due to its simple biology. Over 70 genes have been found to influence lifespan in this worm. The majority of the genes could be placed into canonical longevity pathways including insulin/IGF, germline, TOR signaling, and mitochondrial respiration. The insulin/IGF-1 signaling (IIS) pathway is a central regulator of life span. Transcription factors regulated by the IIS and the TOR-S6K pathways are implicated in the anti-aging effects of Dietary restriction (DR). Lifespan extension via germline removal depends on at least four signaling mechanisms: reduced TOR signaling, DAF-16/FOXO regulation, increased steroid signaling via the DAF-36/DAF-9/DAF-12 pathway, and increased NHR-80/HNF-4 signaling. The mitochondrial unfolded protein response (UPRmt), a mitochondrial stress response, can be regarded as a hormetic mechanism that extends lifespan in spite of mitochondrial dysfunction. Longevity- promoting compounds such as rapamycin and resveratrol also induce mitonuclear imbalance that activates the UPRmt.

|  |  |  |
| --- | --- | --- |
| Reference pathway | 184% 150% 122% 100% 82% 67% 55% | 图片下载 |
